# Systematic Study of Preparing Porous CaCO_3_ Vaterite Particles for Controlled Drug Release

**DOI:** 10.3390/nano15161227

**Published:** 2025-08-12

**Authors:** Nan Zhang, Binhang Zhao, Pan Yang, Haifei Zhang

**Affiliations:** Department of Chemistry, University of Liverpool, Liverpool L69 7ZD, UK; nan.zhang@liverpool.ac.uk (N.Z.); binhang.zhao@liverpool.ac.uk (B.Z.); pan.yang@liverpool.ac.uk (P.Y.)

**Keywords:** porous CaCO_3_ particles, vaterite nanoparticles, curcumin, drug release

## Abstract

Porous CaCO_3_ vaterite particles have been widely used as drug carriers for biomedical applications due to their high biocompatibility and low production costs. However, controlling the particle size and porosity of CaCO_3_ nanoparticles with the desired crystalline phase is still challenging. In this study, we have systematically investigated the preparation of CaCO_3_ nanoparticles under various conditions including precursor types/ratios/concentrations, additive concentrations (ethylene glycol), and temperatures. The materials were fully characterized by optical microscopy, scanning and transmission electron microscopy, infrared spectroscopy, powder X-ray diffraction, dynamic laser scattering, thermogravimetric analysis, and gas sorption. The impacts of the reaction parameters were rationalized and the mechanism for the formation of porous vaterite particles was suggested. It was possible to produce porous vaterite nanoparticles (200 nm) under the optimized conditions, which were further used as drug carrier to upload a model drug curcumin. The potential of using these vaterite particles for controlled drug release was demonstrated.

## 1. Introduction

The design and development of suitable drug nano-carriers are essential for controlled drug release. As one of the mostly used inorganic nano-carriers, calcium carbonate (CaCO_3_) has attracted significant attention due to its excellent biocompatibility, low toxicity and degradability in vivo [1,2,3]. CaCO_3_ has three polymorphs: calcite, aragonite, and vaterite, in the decreasing order of thermodynamic stability [4,5,6]. The synthesis of CaCO_3_ particles with controlled polymorphs has been going on for the past decades and continuously attracting extensive research interests [7,8,9]. Among them, vaterite particles show more potential in biomedical applications compared to other two polymorphs, particularly for drug delivery [2,10,11]. Vaterite particles can be synthesized in desirable sizes, ranging from nanometres to micrometres. These particles consist of individual primary nanocrystalline particles ranging from 10 to 30 nanometres, which contributes to its large specific surface area and porous structure within synthesized vaterite secondary particles. Furthermore, vaterite is more hydrophilic and can be rapidly degraded under mild conditions, making it an ideal candidate for drug delivery systems [2,10,11].

The porous structure of vaterite secondary particles allows the uploading of different molecules, making it particularly suitable for the delivery of drugs, proteins and genes [2,10,11]. It has been a challenge to synthesize the less stable vaterite particles. There have been great efforts in synthesizing porous vaterite particles with the focus on particle crystallinity and sizes, e.g., by the use of additives (e.g., polyols, surfactants, etc.) and different reaction systems [9,12,13], varied salt concentrations and reaction temperature [14,15], energy transfer via different mixing methods [16], diffusion control in a hydrogel [17], mechanochemical synthesis [18], and accelerated synthesis enhanced by machine learning [19]. The loading of drug molecules into porous vaterite particles can be achieved by physical adsorption or co-precipitation. Vaterite particles can be used not only as drug carriers alone, but also as part of composite materials (such as hydrogels, implants) to enhance their mechanical properties and therapeutic properties [20]. Vaterite can be transformed into calcite crystals by dissolution-reprecipitation in different media, and even completely dissolved or absorbed. This transformation enables the release of the loaded drug, which is affected by environmental conditions (such as pH, temperature, ionic strength) and external control factors [2,10,20].

In this study, we have systematically investigated the synthesis of calcium carbonate particles, with different calcium sources (CaCl_2_ and Ca(NO_3_)_2_) and carbonate salts (Na_2_CO_3_ and NaHCO_3_). The reaction conditions, including concentration, temperature, concentration of additive (ethylene glycol), and molar ratio of Ca^2+^:CO_3_^2−^, were studied, with the aim to produce vaterite secondary nanoparticles with controllable sizes and surface areas. These particles were fully characterized by various techniques including electron microscopy, powder X-ray diffraction (PXRD), Fourier transform infrared spectroscopy (FTIR), gas sorption, and dynamic laser scattering (DLS). Based on these findings, two types of CaCO_3_ nanoparticles with different crystallinity and porosity were prepared and loaded with a model drug curcumin (a hydrophobic drug with anti-cancer effects [21,22,23]) and a controllable release of curcumin was demonstrated. Both primary nanoparticles and agglomerated secondary particles/nanoparticles are frequently mentioned. To avoid confusion, unless ‘primary nanoparticles’ is specifically mentioned, the general mentioning of particles or nanoparticles refers to the agglomerated secondary particles.

## 2. Materials and Methods

### 2.1. Materials

Sodium bicarbonate (NaHCO_3_) and calcium nitrate tetrahydrate (Ca(NO_3_)_2_·4H_2_O) were purchased from BDH Laboratory and Scientific Equipment (Poole, England). Sodium carbonate (NaCO_3_), ethanol, phosphate buffered saline (PBS) tablets, calcium chloride (CaCl_2_), and curcumin (curcuma longs (Turmeric)) were purchased from Sigma-Aldrich (Gillingham, UK). Ethylene glycol (EG) was purchased from Fluorochem (Hadfield, England). All solvents used were of analytical grade. All chemicals were used as received without any further purification.

### 2.2. Preparation of CaCO_3_ Particles

Different concentrations and ratios of CaCl_2_/Ca(NO_3_)_2_ and NaHCO_3_/Na_2_CO_3_ and different reaction conditions (including the addition of ethylene glycol as an additive) were employed to synthesize CaCO_3_ particles. Table 1 lists the reaction conditions used in this study.

For the synthesis procedure, calcium precursors were simply mixed with carbonate salts with and without EG. The reactions with the presence of EG were stirred at 1200 rpm at 0 °C (in an ice bath), 20 °C, and 60 °C for 30 min. The reactions without EG were stirred at 1200 rpm in an ice bath (0 °C) or room temperature (20 °C) for 10 min. The resulting CaCO_3_ nanoparticles were then collected using membrane vacuum filtration with a 400 nm Whatman filter paper, washed with ethanol to remove EG, and dried at 30 °C for 2 h in a vacuum oven.

After this, two vaterite CaCO_3_ nanoparticles samples (Ca 1 and Ca 2) were prepared for the uploading of curcumin. For the synthesis of Ca 1, 20 mL of 0.5M Na_2_CO_3_ aqueous solution (10:10 (*v*/*v*) water/EG solution) was mixed with 20 mL of 0.5 M Ca(NO_3_)_2_ aqueous solution (10:10 (*v*/*v*) water/EG solution). The reaction was stirred at 1200 rpm for 30 min at room temperature (20 °C). The resulting CaCO_3_ nanoparticles were then collected using membrane vacuum filtration with a standard Whatman filter paper, washed with ethanol, and dried at 30 °C for 2 h.

For the synthesis of CaCO_3_ nanoparticles (vaterite Ca 2), 3 mL of 0.025 M NaHCO_3_ in a 3:17 (*v*/*v*) water/EG) solution was mixed with 3 mL of 0.025 M CaCl_2_ in a 3:17 (*v*/*v*) water/EG solution. The reaction was stirred at 1200 rpm for 1 h at room temperature (20 °C). The resulting CaCO_3_ nanoparticles were then collected using membrane vacuum filtration with a 200 nm Whatman filter paper, washed with ethanol, and dried at 30 °C for 2 h.

### 2.3. Uploading of Curcumin into CaCO_3_ Particles

The loading of curcumin into CaCO_3_ particles (samples Ca 1 and Ca 2) was conducted via a rotary evaporation method. 30 mg of curcumin was first dissolved in 5 mL of ethanol in a 15 mL glass vial. 100 mg of CaCO_3_ particles was subsequently added and suspended in the curcumin solution. The organic solvent (ethanol) was removed using a rotary evaporator, operating at 100 rpm. The water bath was preheated to 45 °C, and the initial pressure was set to 500 mbar. The pressure was gradually reduced to 15 mbar over a period of 4 h and the pressure was maintained at this level until all the solvent was completely evaporated. After the rotary evaporation, the curcumin-loaded CaCO_3_ particles were further dried in a vacuum oven at room temperature for 24 h to ensure complete drying. Once the drying process was completed, the particles were stored in a desiccator to avoid moisture exposure. This is because the vaterite CaCO_3_ particles are sensitive to humidity and require dry conditions for storage.

### 2.4. Drug Release Study

The release study was performed in a 40 mL 1:1 volume ratio of ethanol and phosphate-buffered saline (PBS, pH 7.4) solution at room temperature in centrifuge tubes. Samples were placed in centrifuge tubes on an open-air shaker. At predetermined time points, the sample tubes were centrifuged at 12,000 rpm for 4 min. Following centrifugation, 0.4 mL of the PBS-ethanol solution was removed and replaced with an equal volume of fresh solution. The collected PBS ethanol solution was analysed using a Varioskan LUX Multimode Microplate Reader (Thermo Scientific, Waltham, MA, USA) at a wavelength of 429 nm to measure the release of curcumin.

### 2.5. Characterization

The morphology and porous structure of CaCO_3_ particles were investigated by optical microscopy (OM), scanning electron microscopy (SEM), and transmission electron microscopy (TEM). For OM, samples were placed on the microscope slide and then covered with a cover slip. The amorphous-to-crystalline phase transformation of CaCO_3_ were observed using an Olympus CX41-Met Upright Microscope (Olympus, Southend-on-Sea, UK). For SEM, CaCO_3_ particles were placed on the carbon sticker which was stuck to a 12 mm diameter aluminum sample holder. Conductive sliver paint (Agar Scientific Ltd., Rotherham, UK) was applied around the carbon sticker, followed by sputter coating with a gold layer (4 nm). The morphology was then examined by a Hitachi S-4800 SEM (Hitachi Europe Ltd., Berkshire, UK). For TEM, the images were taken using a JEOL 2100+ TEM (Welwyn Garden City, UK) operating at 200 KV. The size of CaCO_3_ particles in a suspension was measured by dynamic light scattering (DLS) using Zetasizer Nano ZS (Malvern Instruments Ltd., Malvern, UK), with water as the dispersant at 25 °C.

A Micrometric 3-Flex 3500 Gas Sorption Analyser (Malvern Panalytical, Malvern, UK) was used to measure surface area and the pore sizes. A weighed sample (0.1–1.0 g) was loaded into a measuring tube, connected to the instrument, and degassed at 100 °C for 4 h. Gas sorption measurements with N_2_ were then conducted at 77.3 K. After analysis, the sample was re-weighed for subsequent calculations of surface area, pore volume, and pore diameter.

The thermal behaviour of the samples was determined by thermal gravimetric analysis (TGA) using Netzsch TG 209 F1 Libra (Netzsch Thermal Instruments Ltd., Wolverhampton, UK). Weighed (1.0–3.0 mg) samples were loaded into aluminum crucible and heated from 0 to 200 °C at a heating rate of 10 °C/min; N_2_ gas was used for purging. The data was analysed using NETZSCH Proteus Thermal Analysis (Netzsch Thermal Instruments Ltd., Wolverhampton, UK).

X-ray diffraction patterns for CaCO_3_ particles were recorded on a Bruker-D8 ADVANCE X-Ray diffractometer (Bruker, Brighton, UK). with Cobalt radiation. The scans were collected over 5−40° 2 theta (2θ) range with a step size of 0.5° 2θ and a scan speed of 1 s.

Fourier transform infrared spectroscopy (FTIR) spectra were obtained by using a FT-IR Vertex 70 spectrometer (Bruker, Brighton, UK). The spectra were recorded under the attenuated total reflection (ATR) mode.

## 3. Results and Discussion

Different methods have been reported to produce CaCO_3_ particles [3,8,12,13,14,15,16,17,18,19]. The precipitation method via the mixing of soluble Ca^2+^ salts and CO_3_^2−^/HCO_3_^−^ salts is effective, fast, and can be easily scaled up. However, to fabricate the metastable vaterite CaCO_3_ particles, dilute concentrations, low temperature, short reaction time, and the addition of additives (e.g., alcohols, diols, surfactants, polymers) have been utilized in the synthesis. However, the challenge in preparing nanosized vaterite particles with tuneable morphology and porosity remains. Ethylene (EG) is one of the mostly used additives because it is readily available, miscible in water, and can be easily used to prepare salt solutions [3,8,12,14]. In this study, we have systematically investigated different reaction parameters, with EG as the additive. This allowed the optimization of reaction conditions for the preparation of small porous vaterite nanoparticles with adjustable surface area and pore size, which were then used as carrier for the uploading and release of curcumin.

### 3.1. Synthesis of CaCO_3_ Particles with Varying Contents of EG

**Figure 1:**
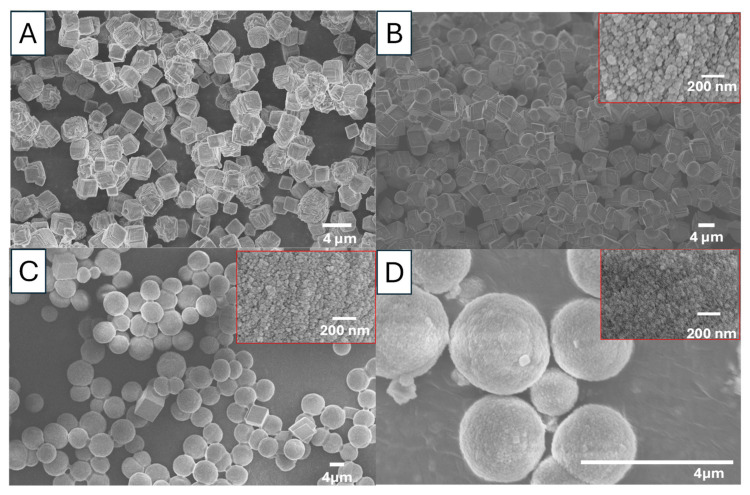
SEM images of CaCO_3_ prepared by rapid mixing of a 0.5 M Na_2_CO_3_ solution with a 0.5 M CaCl_2_ solution at room temperature (20 °C) in the presence of EG: (**A**) no EG (S1). (**B**) 15 *v*/*v*% EG (S2). (**C**) 50 *v*/*v*% EG (S3). (**D**) 85 *v*/*v*% EG (S4).

**Figure 2:**
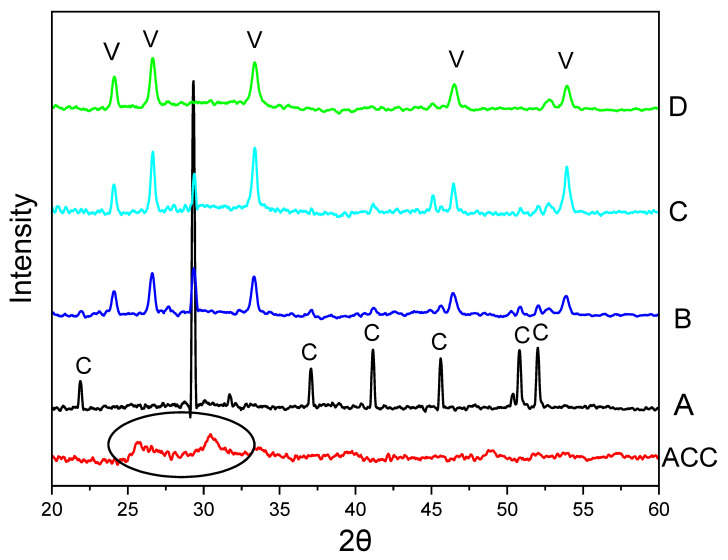
PXRD diffractograms of CaCO_3_ particles prepared by rapid mixing of a 0.5 M Na_2_CO_3_ solution with a 0.5 M CaCl_2_ solution at room temperature (20 °C) in the presence of EG: (A) no EG (S1). (B) 15 *v*/*v*% EG (S2). (C) 50 *v*/*v*% EG (S3). (D) 85 *v*/*v*% EG (S4). (ACC) Initial precipitate amorphous calcium carbonate (ACC) with the black circle indicating lack of distinct crystalline peaks. V indicates the peaks for vaterite phase while C depicts the peaks for calcite phase.

The reactions were first carried out between Na_2_CO_3_ and CaCl_2_ with a concentration of 0.5 M and EG concentrations of 0, 15, 50, and 85 *v*/*v*%. Figure 1 presents the SEM images of CaCO_3_ particles formed under different concentrations of EG. These images demonstrate a morphology change of cubic particles to spherical particles, when the concentration of EG was increased from 0 *v*/*v*% to 85 *v*/*v*%. Figure 2 shows the PXRD patterns of CaCO_3_ particles, illustrating a crystalline phase change of calcite, mixture of calcite and vaterite, and vaterite particles. In Figure 1A and Figure 2A, there was no EG involved in the reaction (sample S1, Table 1), only calcite particles were formed. However, upon the addition of EG (15 *v*/*v*%, 50 *v*/*v*%, 85 *v*/*v*%), vaterite particles began to appear and then dominate in the precipitates (Figure 1B–D and Figure 2B–D). Indeed, when the EG concentration reached 85 *v*/*v*%, no calcite was observed, and no calcite peaks appeared in the PXRD diffractogram. This indicates that at this concentration, the ratio of water to EG is optimal for promoting and maintaining vaterite formation.

The initially precipitated slush was also characterized by PXRD. It exhibited no sharp peaks in the PXRD diffractogram, indicating a lack of crystalline structure and confirming the formation of amorphous calcium carbonate (ACC) (circled in the diffractogram for ACC in Figure 2). The PXRD pattern in Figure 2A reveals peaks at 2θ values of 21.90°, 29.31°, 37.06°, 41.16°, 45.68°, 50.83°, and 52.04°, corresponding to the crystallographic planes (012), (104), (110), (013), (202), (018), and (116). These peaks confirm that the CaCO_3_ particles are calcite. The peak at 29.31°, corresponding to the (104) crystallographic plane, is the main peak for calcite. In contrast, the PXRD diffractogram for Figure 2D shows diffraction angle 2θ at 24.10°, 26.69°, 33.32°, 46.56°, and 53.9°, corresponding to the crystallographic planes (100), (101), (102), (110), (104), and (202), indicating that the particles formed are vaterite [5,6,24]. With the introduction of EG for the synthesis of the sample shown in Figure 2B, the intensity of the calcite peak at 29.3° reduces while vaterite peaks intensity start to appear and increase (Figure 2C).

**Figure 3:**
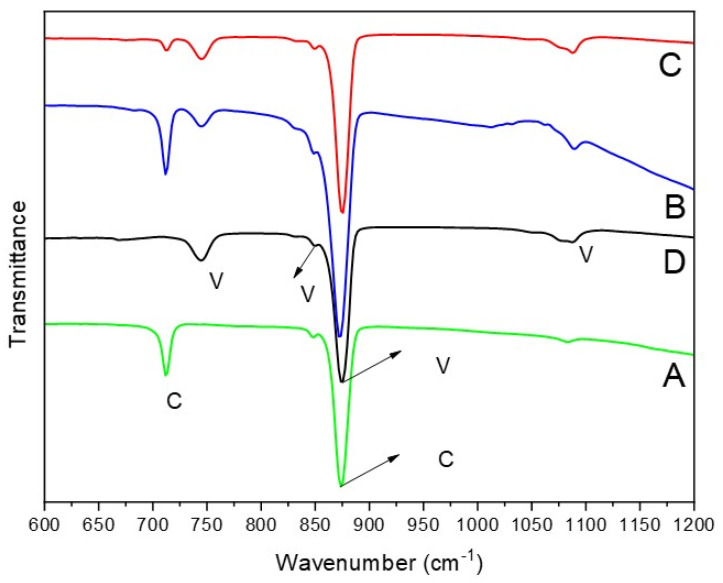
FTIR spectra of CaCO_3_ prepared by rapid mixing of a 0.5 M Na_2_CO_3_ solution with a 0.5 M CaCl_2_ solution at room temperature (20 °C) in the presence of EG: (A) no EG (S1). (B) 15 *v*/*v*% EG (S2). (C) 50 *v*/*v*% EG (S3). (D) 85 *v*/*v*% EG (S4). V indicates the peaks for vaterite phase while C depicts the peaks for calcite phase.

The polymorphs of CaCO_3_ particles can also be confirmed by FTIR analysis [24,25]. In general, the FTIR spectra of CaCO_3_ exhibit four absorption bands corresponding to carbonate ions, which has different vibrations modes: symmetric stretching (ν_1_), out-of-plane bending (ν_2_), doubly degenerate asymmetric stretching (ν_3_), and doubly degenerate in-plane bending (ν_4_) [25]. In Figure 3, the FTIR results indicate that two strong peaks are observed in the synthesized calcite particles (Figure 3A), appearing at 712 cm^−1^ (in-plane (ν_4_)) and 876 cm^−1^ (out-of-plane(ν_2_)). In comparison, as shown in Figure 3D, the FTIR peaks of vaterite are detected at 745 cm^−1^ (an in-plane bending ν_4_), 849 cm^−1^, 877 cm^−1^ (out-of-plane bending ν_2_), and 1088 cm^−1^ (a symmetric carbonate stretching band ν_1_). Notably, an overlapping absorption peak of vaterite and calcite is out-of-plane bending (ν_2_) vibrational, which also exists in aragonite and was recorded in the 876–877 cm^−1^ range in this study. For the samples in Figure 3B,C, the FTIR spectra show peaks corresponding to both calcite and vaterite, indicating a mixture of both polymorphs. Particularly, the FTIR spectrum in Figure 3B exhibits a higher intensity at 712 cm^−1^, which corresponds to the calcite abdorption band, suggesting a greater amount of calcite in the sample. This observation is consistent with the findings in Figure 1 and Figure 2.

**Figure 4:**
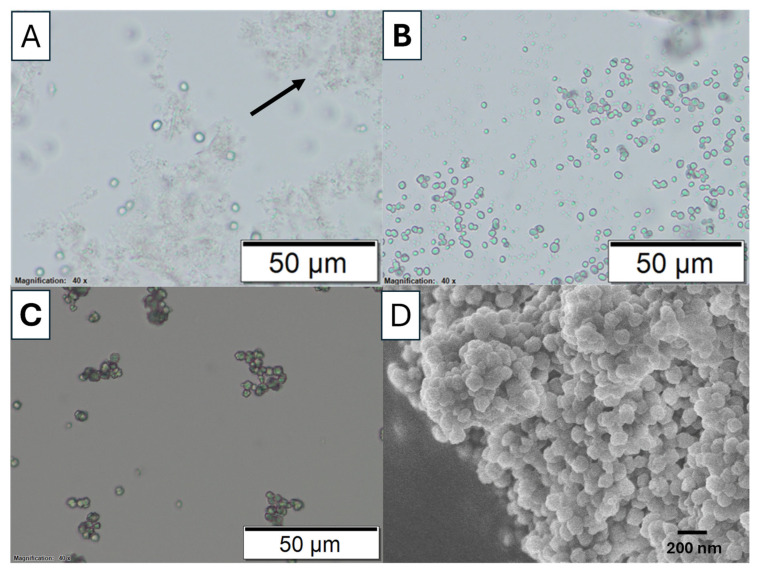
Microscopic images of calcium carbonate phase transformation, with Na_2_CO_3_ solution (0.5 M) rapidly mixing with CaCl_2_ solution (0.5 M) in the ice bath (sample S5). The microscopic images were taken at (**A**) 1 min. The arrow highlights the ACC formed. (**B**) 10 min and (**C**) 60 min. (**D**) SEM image of amorphous calcium carbonate.

The phase transformation of calcium carbonate (CaCO_3_) from the amorphous state to crystalline calcite state was studied via optical microscopy. Figure 4 shows the optical microscopic images of this transformation when the reaction was carried out in an ice bath (to slow down the reaction) without EG added. After mixing calcium ions (Ca^2+^) and carbonate ions (CO_3_^2−^), the transition from an ACC phase to a crystalline phase was observed at different time points of 1, 10, and 60 min. Figure 4A shows only a few nuclei are visible after 1 min. Most of the observed particles appear to be a gel-slushie state. At this stage, the precipitation process is dominated by the formation of ACC. ACC is an unstable, highly hydrated polymorph that lacks the defined crystalline structure which normally transforms into the other polymorph within serval minutes [26]. According to Konrad et al., ACC begins to dissolve when the humidity reaches a certain level. As ACC starts to dehydrate, the released water increases the surrounding moisture, leading to its dissolution and followed by recrystallization [27].

At 10 min, as shown Figure 4B, more crystals are formed, indicating the ongoing transformation of ACC into a more stable crystalline phase. No significant ACC slush was observed. For the reaction at 60 min, barely any other phases are visible under an optical microscope, and the particle sizes appear to be more uniform (Figure 4C). This suggests that most likely all the ACC has converted into a single crystalline phase. The uniform particle size indicates a reduction in nucleation as the dehydrated ACC is consumed. With fewer new nuclei forming, crystal growth becomes more prominent, leading to the growth of larger calcite crystals. The uniformity in particle size at this stage can be attributed to Ostwald ripening, a process in which smaller particles dissolve and redeposit onto larger calcite crystals, promoting their growth. FTIR spectra further confirm that most of the particles in Figure 4B,C are calcite (Figure S1). The morphology of the amorphous calcium carbonate collected after 5 min in the reaction is revealed by SEM in Figure 4D, which shows agglomerated spherical particles and are distinctly different from the calcite particles.

It is clear that the formation of vaterite particles is a result of increasing concentrations of EG in the reaction system. The hydroxyl groups offered by EG could play an important role in vaterite formation. EG could act as either a co-surfactant or a co-solvent depending on the concentration in the reaction system. At low concentrations, EG functions as a co-surfactant with the negative charge presented by the hydroxyl groups. After the initial formation of vaterite nuclei, EG molecules are attracted to the vaterite nuclei. The negative charge of these hydroxyl groups could prevent the transformation of vaterite into calcite [7]. Additionally, the presence of hydroxyl groups alters the surface charge of vaterite, making it more thermodynamically stable compared to calcite in the system [12]. This impact is enhanced with the increasing concentration of EG. At higher concentrations of EG, the abundance of hydroxyl groups and free Ca^2+^ ions favor the nucleation of vaterite. Calcium precursor salts dissolve in both water and EG, whereas carbonates primarily dissolve in water. As the concentration of EG increases, the solubility of carbonate salts decreases [12]. As a result, the supersaturation of carbonate salts is higher than that of calcium ions, further promoting vaterite formation. The concentration of Ca^2+^ could influence CaCO_3_ growth. It was reported that the increased concentration of Ca^2+^ led to the reduced particle size [28]. As a result, EG can act as both a stabilizer, preventing the transformation of vaterite into other polymorphs, and a promoter, enhancing vaterite formation. Therefore, as the concentration of EG increases, vaterite particles are produced, whereas calcite dominates the formation in the absence of EG.

Another effect from the addtive is the viscosity of the reaction system, thereby altering nucleation and growth rates [29]. The viscosity of the reaction system affects the diffusion velocity of ions, with the crystallization speed being determined by the diffusion rate of Ca^2+^. An earlier study confirmed that an increase in reaction viscosity contributed to higher diffusion resistance to ions [30]. Higher viscosity inhibits particle growth, causing the nucleation process to dominate. In the presence of EG, the reaction viscosity increases. It is worth noting that the viscosity of polyols is dictated by the number of hydroxyl groups forming hydrogen bonds. The reduced diffusion velocity of Ca^2+^ and CO_3_^2−^ ions could slow nucleation and crystal growth, leading to the formation of smaller crystal [31]. This trend can be observed in Figure 1.

The presence of EG could also impact the supersaturation of the reaction [12]. The supersaturation increases when the concentration of EG in the reaction increases. The supersaturation of the reaction solution is a key factor in the control of the crystallization because it affects the nucleation and growth of the crystal. The supersaturation ratio S can be defined by this equation [29]:

$$S=\frac{\left[{Ca}^{2+}\right]\times \left[{{CO}_{3}}^{2-}\right]}{K_{sp}}$$

where [Ca^2+^] and [CO_3_^2−^] represent the concentrations of calcium and carbonate ions, respectively, and K_sp_ is the solubility product constant of calcium carbonate. When S < 1, the solution is unsaturated. The solution reaches equilibrium when S = 1. When S > 1, calcium carbonate starts to precipitate to restore equilibrium in the solution, which is thermodynamicallly favoured. Increasing supersaturation subsequently influences the size of the primary stucture of the vaterite [4,31,32]. High supersaturation favours the nucleation of the calcium carbonate crystals, leading to smaller individual crystallites as observered when the concentration of the EG was increased. This reduction in crystallite size is attributed to calcium ions associating with the polar -OH groups in EG, which increases the local supersaturation, providing more nucleation sites in the system [24]. Crystals grow faster at a low supersaturation, however, crystal nucleation domionates the growth of the crystal at high supersaturation, leading to smaller primary nanoparticles which agglomerate to form the vaterite particles [31].

**Figure 5:**
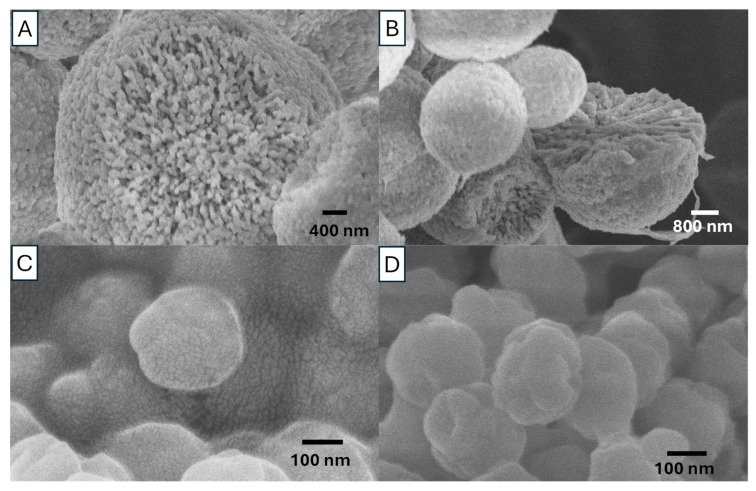
(**A**,**B**) SEM images of internal structure of broken vaterite particles, prepared by rapid mixing of 0.5 M Na_2_CO_3_ solution and CaCl_2_ solution with an EG content of 85 *v*/*v*% at 20 °C (S6). (**C**,**D**) SEM images of the precipitate ACC before the formation of vaterite.

Figure 5 shows the internal structure of vaterite particles after mechanical fracture, prepared from 0.5 M solutions with an EG concentration of 85 *v*/*v*% at 20 °C. The SEM images indicate that the vaterite synthesized in this study is formed through the agglomeration of nanocrystals. The vaterite particles exhibit a highly interconnected internal structure, composed of agglomerated layer-by-layer nanoparticles. Based on our observations in Figure 4 and Figure 5 and literature reports [28,29,30,31,32], we suggest that the growth of porous vaterite particles is likely to follow this mechanism. First, when calcium and carbonate salts react, ACC precipitates to form a slush-like crystalline state. Then the ACC loses water, and it transforms into a more ordered, homogeneous nanocrystalline structure. These nanocrystals then rapidly aggregate, forming a porous polycrystalline structure, which represents the secondary structure of vaterite particles. In the final step, following Ostwald ripening, larger porous vaterite particles are formed, with all these steps facilitated by the presence of EG.

### 3.2. Effect of Concentration

**Figure 6:**
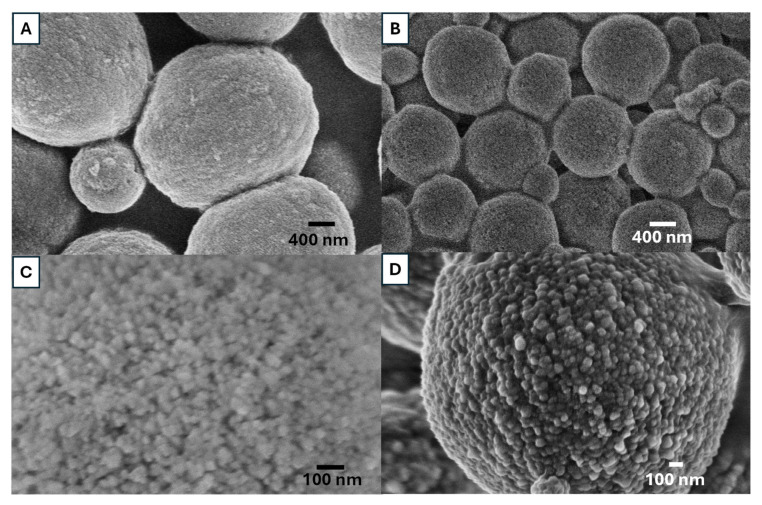
SEM images of vaterite CaCO_3_ particles prepared by rapid mixing of 0.1 M Na_2_CO_3_ solution (85 *v*/*v*% EG) with a 0.1 M CaCl_2_ solution (85 *v*/*v*% EG), at (**A**,**C**) 20 °C (Sample S7) and (**B**,**D**) 60 °C (Sample S8).

**Figure 7:**
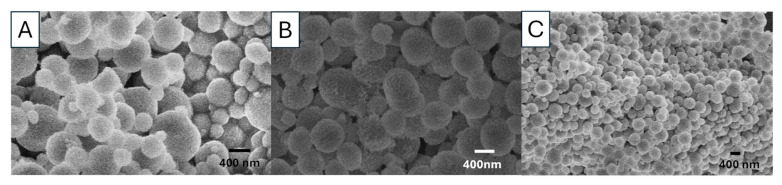
SEM images of vaterite CaCO_3_ particles prepared by rapid mixing of 0.05 M Na_2_CO_3_ solution (85 *v*/*v*% EG) with a 0.05 M CaCl_2_ solution (85% *v*/*v*% EG), at (**A**) 0 °C (sample S9), (**B**) 20 °C (sample S10) and (**C**) 60 °C (sample S11).

**Figure 8:**
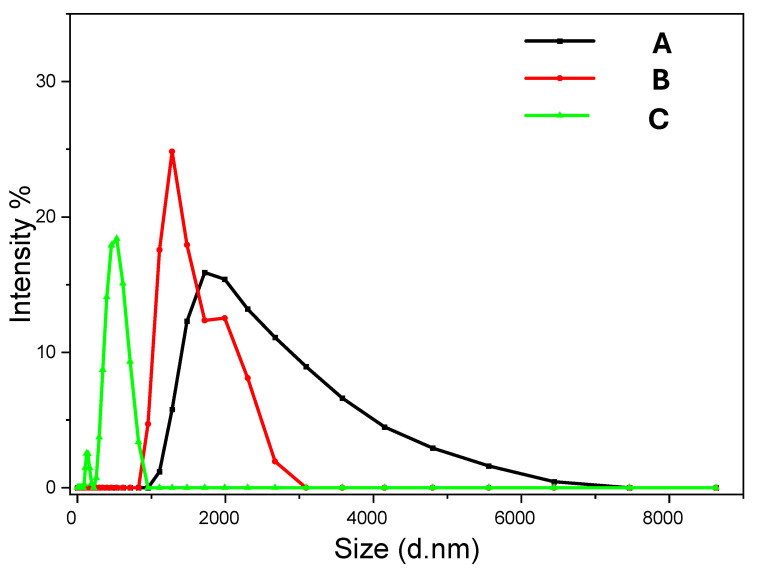
The size distribution of vaterite particles measured by DLS. (A) 0.5 M Na_2_CO_3_ solution + 0.5 M CaCl_2_ solution with 85 *v*/*v*% EG at 20 °C. (B) 0.1 M Na_2_CO_3_ solution + 0.1 M CaCl_2_ solution with 85 *v*/*v*% EG at 20 °C. (C) 0.05 M Na_2_CO_3_ solution + 0.05 M CaCl_2_ solution with 85 *v*/*v*% EG at 20 °C.

Our investigation started with the concentration of 0.5 M Na_2_CO_3_ and CaCl_2_ solutions. Lower concentrations are expected to slow down the nucleation and crystal growth. With an aim to produce smaller vaterite particles, the concentrations were reduced to 0.1 M and 0.05 M with the same EG concentration of 85 *v*/*v*% and reaction time at room temperature (20 °C). Figure 6A shows the vaterite particles prepared from 0.1 M solutions while the particles prepared from 0.05 M solutions are given in Figure 7B. Compared to the vaterite particles prepared from 0.5 M solutions (Figure 1D), it is clear that the diameter of the vaterite particles decreases with the decreasing concentration of the precursors. This has been further confirmed by measuring the particles suspensions with DLS. Figure 8 presents the particle size distribution of vaterite particles prepared from three different precursor concentrations. The size distribution is rather broad for particles prepared with higher concentrations (e.g., 0.5 M) but becomes narrower when the solution concentration is reduced. The particle sizes at the peak position in Figure 8 are around 2 µm (prepared from 0.5 M solutions), 1.5 µm (from 0.1 M solutions), and 600 nm (from 0.05 M solutions).

**Figure 9:**
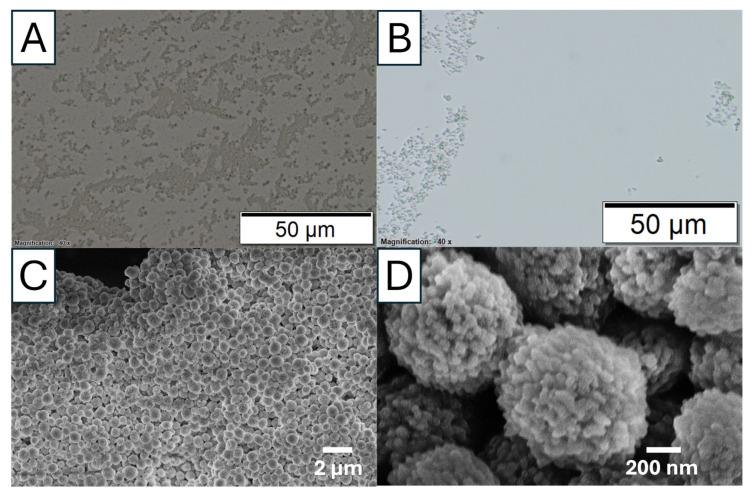
Optical microscopic images and SEM images showing the phase transformation from ACC to vaterite, via a reaction of 0.05 M Na_2_CO_3_ solution + 0.05 M CaCl_2_ solution with 85 *v*/*v*% EG at 20 °C. The microscopic images were taken at (**A**) 10 min (ACC and vaterite). (**B**) 30 min (vaterite particles). (**C**,**D**) SEM images of the secondary vaterite nanoparticles at different magnifications.

The phase transformation of calcium carbonate from ACC to vaterite has been investigated by optical and SEM imaging (Figure 9). Different mechanisms have been proposed to explain this phase transition [5,6,28,29,30,31,32]. The more plausible mechanism, as observed in Figure 9, is that ACC first dissolves, leading to the homogeneous nucleation of primary vaterite nanoparticles. These nanocrystalline particles then rapidly aggregate, forming a porous polycrystalline secondary structure. Figure 9D highlights the morphology of secondary vaterite nanoparticles, consisting of agglomerated primary nanocrystalline particles.

### 3.3. Effect of Reaction Temperature

Reaction temperature can significantly affect nucleation rate, grain growth and final grain size [33,34]. Figure 6 shows the vaterite particles prepared at room temperature (20 °C) and 60 °C from 0.1 M solutions with 85 *v*/*v*% EG. A notable size decrease is observed (Figure 6A,B). These vaterite particles consist of numerous individual but agglomerated primary nanoparticles. Feoktistova et al. found that the size of primary nanoparticles could be affected by reaction temperature; when the temperature increased, the size of primary nanoparticles increased [35]. The crystallization of vaterite begins with the formation of ACC when Ca^2+^ and CO_3_^2−^ ions are mixed. ACC has been comfirmed as CaCO_3_·H_2_O, is thermodynamically unstable and crystallizes at ambient temperatures, leading to the formation of calcium carbonate and its polymorphs. The spherulitic growth of vaterite proceeds via a three step process [35,36]. First, ACC loses the water to a dehydrated form, transitioning into a more ordered nanocrystalline structure, known as the primary structure of vaterite. Second, the nanocrystalline particles start to grow because the supersaturation of the vaterite is low, where smaller particles dissolve, promoting nanocrystallite growth. The final steps is dominated by the surface-mediated Ostwald ripening rule [37]. At a higher temperature, faster reaction rates accelerate Ostwald ripening, resulting in larger nanocrystallites compared to lower reaction temperatures [35,36]. This is consistent with our findings, as shown in Figure 6C,D. When the reaction temperature was 60 °C, the sizes of the nanocrystallities are less than 50 nm. When the reaction temperature was increased to 60 °C the nanocrystallites sizes are larger than 50 nm.

Figure 7 shows the SEM images of the vaterite particles synthesized by rapidly mixing 0.05 M Na_2_CO_3_ solution and 0.05 M CaCl_2_ with 85 *v*/*v*% EG at three different temperatures (0, 20, and 60 °C). The vaterite particle surface appears rough but becomes progressively smoother as the temperature increases. The SEM images show that higher temperatures lead to the formation of more uniform but smaller particles. All vaterite particles maintain a spherical shape. The descrease in particle sizes is consistent with the observation of vaterite particles prepared from 0.1 M solutions at different temperatures.

To anyalse the porosity of the secondary vaterite particles, N_2_ sorption was used to measure the pore size and the specific surface area by the Brunauer–Emmett–Teller (BET) method at 77.3 K. Pore diameters were determined using the Barrett–Joyner–Halenda (BJH) analysis based on the desorption isothermal curves. The results are presented in Table 2. The specific surface areas of these samples are influenced by the synthesis temperature. When the temperature increases from 0 °C to 20 °C, the specific surface area increases. However when the temperature increase from 20 °C to 60 °C, the specific surface area decreases. It is also observed that pore diameter and pore volume consistently increase with the increase of reaction temperature. This may be explained by the increasing size of individual primary nanocrystals (which aggregate to form the secondary spherical particles) at higher reaction temperatures. This can reduce the surface-to-volume ratio and result in larger interstitial voids formed from the agglomerated primary nanocrystalites, with some pores larger than 50 nm (Figure S2).

### 3.4. Effect of Reaction Precursors and the Ratio of Ca^2+^:CO_3_^2−^

**Figure 10:**
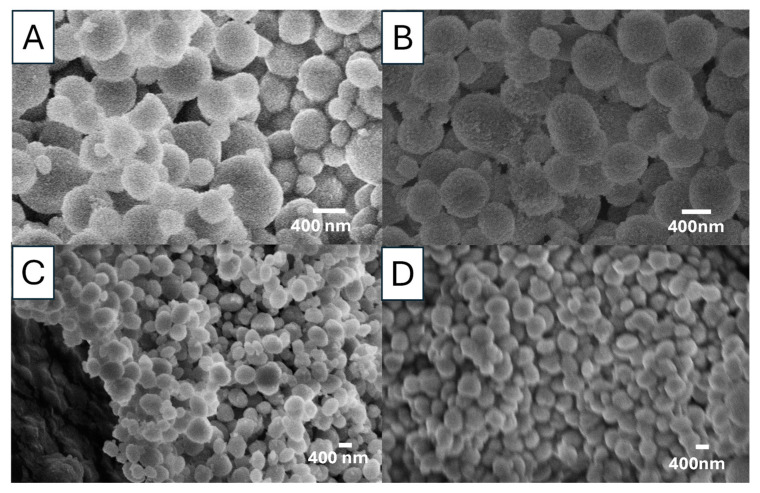
The SEM images of the morphology of vaterite particles formed at a Ca^2+^:CO_3_^2−^ ratio of 1:1. (**A**) 0.05 M of Na_2_CO_3_: Ca(NO_3_)_2_ at 20 °C (S12). (**B**) 0.05 M of CaCl_2_:Na_2_CO_3_ at 20 °C (S10). (**C**) 0.05 M of Ca(NO_3_)_2_, NaHCO_3_ at 20 °C (S13). (**D**) 0.05 M of CaCl_2_, NaHCO_3_ at 60 °C (S14).

We further examined the effect of different Ca^2+^:CO_3_^2−^ ratios (1:1 and 1:2) with different reaction precursors (Na_2_CO_3_ and Ca(NO_3_)_2_) on the formation of vaterite particles. Indeed, all the reactions conducted so far were using Na_2_CO_3_ and CaCl_2_ solutions with a Ca^2+^:CO_3_^2−^ ratio of 1:1, but under different concentrations and reaction temperatures. At the same Ca^2+^:CO_3_^2−^ ratio of 1:1, different combinations of Ca^2+^ and CO_3_^2−^ precursors were investigated with 85 *v*/*v*% EG at 20 °C and 60 °C (Figure 10). The SEM inages in Figure 10 show that the choice of precursor has no significant impact on the morphology of the vaterite particles. All the particles produced are spherical, although some particles are agglomerated. The particle size distributions look similar but there is a trend that the use of NaHCO_3_ as precursor and higher reaction temperature lead to wider size distribution and smaller particles (Figure S3). The FTIR results (Figure S4) indicate that vaterite particles were formed and the use of different precursors under these reaction conditions did not alter the crystalline vaterite structure.

**Figure 11:**
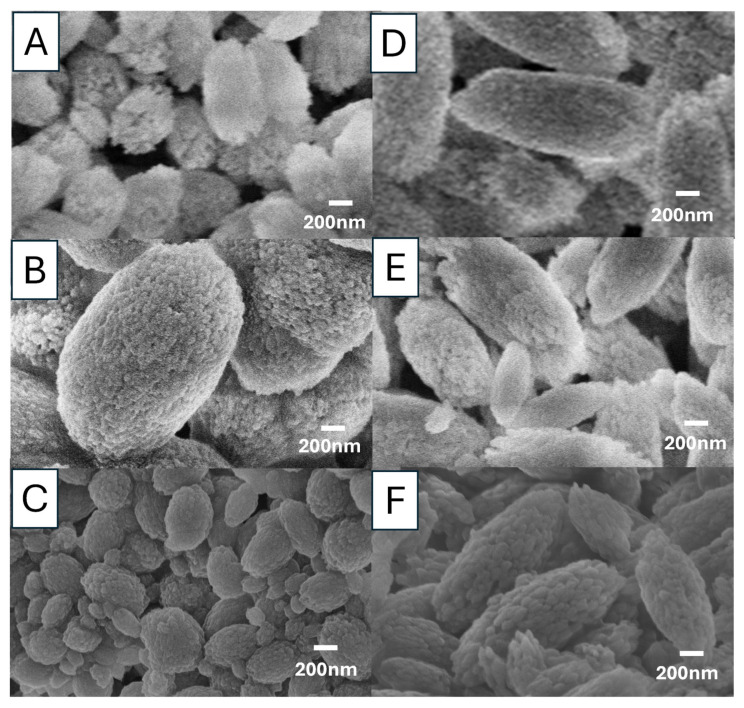
The SEM images show the morphology of vaterite particles formed at a Ca^2+^:CO_3_^2−^ ratio of 1:2 with 85 *v*/*v*%. (**A**) 0.05 M of CaCl_2_, NaHCO_3_ at 20 °C (S15). (**B**) 0.5 M of Na_2_CO_3_, Ca(NO_3_)_2_ at 20 °C (S16). (**C**) 0.05 M of CaCl_2_, Na_2_CO_3_ at 20 °C (S17). (**D**) 0.1 M of CaCl_2_, Na_2_CO_3_ at 60 °C (S18). (**E**) 0.1 M of CaCl_2_, NaHCO_3_ at 60 °C (S19). (**F**) 0.1 M of Ca(NO_3_)_2_, NaHCO_3_ at 60 °C (S20).

For the Ca^2+^:CO_3_^2−^ ratio of 1:2, the reactions were performed at varied concentrations (0.5 M, 0.1 M, and 0.05 M) and temperatures (20 °C, 60 °C) with different precursors. FTIR analysis of these samples (Figure S5) confirmed that vaterite particles had been produced. Figure 11 presents the morphologies of these particles. It is clear that the increase in the molar ratio of carbonate salts has a significant impact on the particle morphology—a change of particle shape from spherical to ellipsoidal. The formation of ellipsoidal vaterite was observed, regardless of the precursor used, precursor concentrations and reaction temperature, at a 1:2 Ca^2+^:CO_3_^2−^ ratio. It is difficult to compare the size of these particles because this study intended to invesigate the effect of precursor/concentration/temperature and the conditions were not directly comparable. However, it is still possible to draw some qualitative conclusions, for example, higher concentrations leading to larger particles (particles in Figure 11B largest, particles in Figure 11D–F generally larger than the paricles in Figure 11A,C). The sizes of primary nanocrystallites (which aggregate to form the secondary spherical structures) are different among the precursors (e.g., Figure 11D vs. Figure 11E). The ellipsoidal particles become more elongated with the increase of reaction temperature.

### 3.5. CaCO_3_ Particles (Ca 1 and Ca 2) for Curcumin Loading

Two types of CaCO_3_ particles (samples Ca 1 and Ca 2 in Table 1) were prepared, with different size, porosity, and crystallinity. Because our objective is to use CaCO_3_ particles for drug loading and release/delivery, nanoparticles with higher porosity are highly desirable. Sample Ca 1 was prepared with 0.5 M Na_2_CO_3_ solution + 0.5 M Ca(NO_3_)_2_ solution with 50 *v*/*v*% EG at room temperature, mainly serving as a control sample. Sample Ca 2 was produced as porous vaterite nanoparticles, under the conditions of 0.025 M NaHCO_3_ + 0.025 M CaCl_2_ with 85 *v*/*v*% EG stirred at 1200 rpm for 1 h at room temperature. This is based on our earlier findings that lower precursor concentration and the use of NaHCO_3_ with high EG content contribute to the formation of smaller vaterite particles.

**Figure 12:**
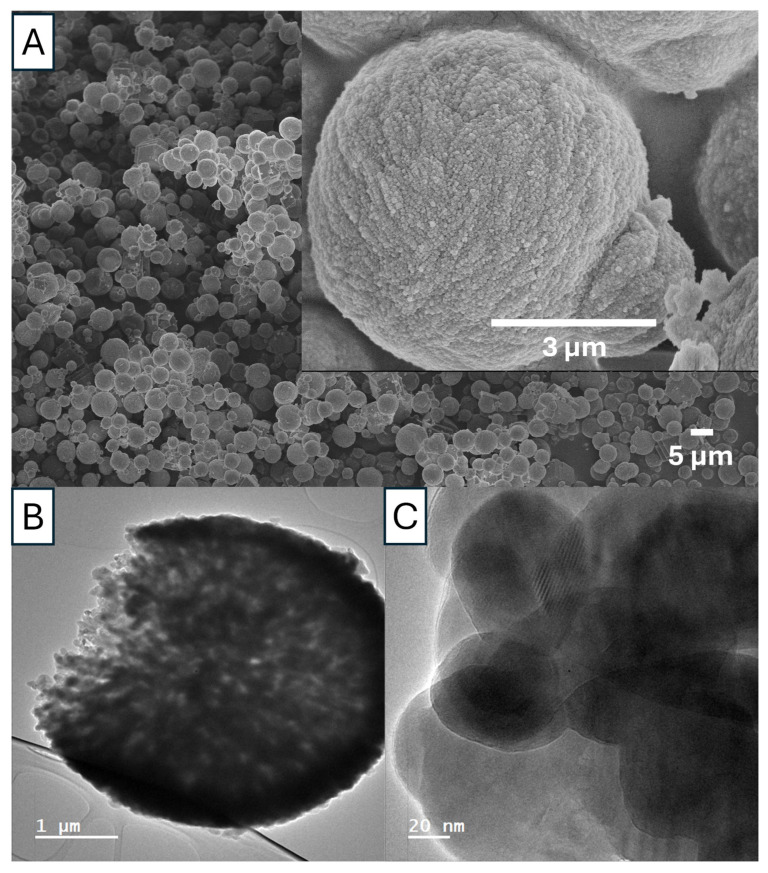
SEM (**A**) and TEM (**B**,**C**) images of CaCO_3_ particles prepared with 0.5 M Na_2_CO_3_ solution + 0.5 M Ca(NO_3_)_2_ solution and 50 *v*/*v*% EG at 20 °C (sample Ca 1).

**Figure 13:**
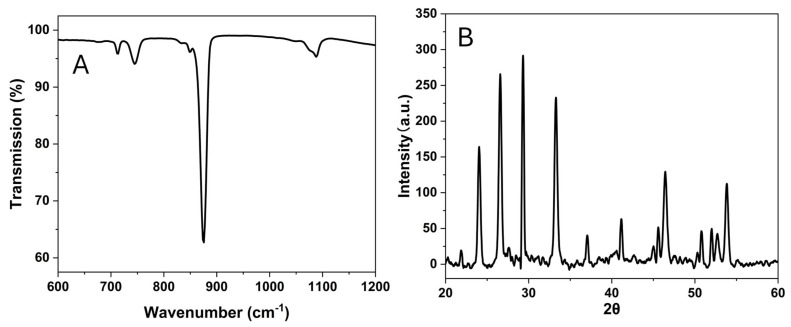
FTIR spectrum (**A**) and PXRD diffractogram (**B**) of sample Ca 1.

As shown in Figure 12 and Figure 13, Ca 1 is a mixture of vaterite and calcite particles with the diameters in the range of 1–5 µm. The porosity and the crystalline structure can be revealed by the TEM images in Figure 12B,C. The FTIR peaks (Figure 13A) indicate that the particles contain both vaterite and calcites. The vibration bands are observed at 713.1 cm^−1^(calcite), 744.5 cm^−1^ (vaterite), and overlapping at 849 cm^−1^, 876 cm^−1^, and 1088 cm^−1^. Additionally, the XRD diffractogram (Figure 13B) shows the diffraction angles at 24.02°, 26.56°, 29.34°, 33.26°, 37.05°, 41.13°, 44.99°, 46.45°, 50.72°, and 53.79°, indicating that the particles are the mixture of vaterite and calcite [24,25].

**Figure 14:**
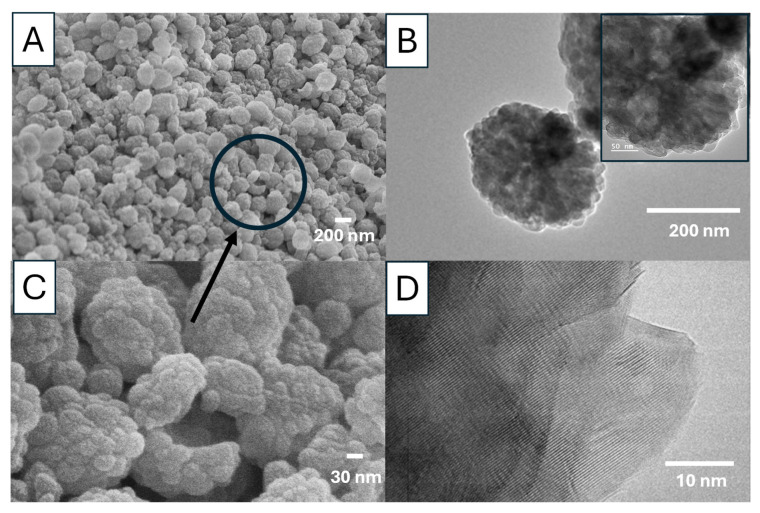
SEM (**A**,**C**) and TEM (**B**,**D**) images of vaterite nanoparticles prepared by rapid mixing of 0.025 M NaHCO_3_ solution + 0.025 M CaCl_2_ solution with 85 *v*/*v*% EG at 20 °C (sample Ca 2).

For sample Ca 2, the vaterite nanoparticles are around 200 nm (Figure 14A,B), consisting of agglomerated primary nanocrystals in the range of 20–40 nm (Figure 14C). The primary nanocrystals are relatively uniform, with most particles around 30 nm (Figure 14B,C). The TEM images show the porous structure of these vaterite particles and the crystalline structure (a Moiré pattern) (Figure 14B,D). The FTIR spectrum with the typical peak at 745 cm^−1^ indicates the formation of vaterite nanoparticles whilst the PXRD pattern with the peaks at the diffraction angles of 23.98°, 26.61°, 33.27°, 46.40°, and 53.79° (corresponding to the crystallographic planes (100), (101), (102), (110), (104), and (202)) further confirms the vaterite crystalline structure (Figure S6) [24,25].

The surface area and porosity of Ca 1 and Ca 2 were evaluated by N_2_ sorption. The specific surface areas of 5.32 m^2^/g and 20.40 m^2^/g were obtained for Ca 1 and Ca 2, respectively (Table 3). The analysis results show higher surface areas, larger pore diameter, and much higher pore volume for Ca 2 (Table 3). This suggests that Ca 2 should have better potential as a drug carrier for higher drug loading.

### 3.6. Loading and Controlled Release of Curcumin

The loading of a model drug curcumin was achieved by soaking Ca 1 and Ca 2 particles in curcumin solutions, followed by rotary evaporation to remove the solvent. Thus, the encapsulation efficiency of Ca 1 and Ca 2 particles were considered to be close to 100%. Based on this assumption, the drug loading capacity was calculated to be 3.1% for both Ca 1 and Ca 2. Curcumin is a hydrophobic polyphenolic compound [21]. As one of the promising drug candidates for cancer treatment, curcumin could be used on its own or associated with other drugs [22,23]. To increase the bioavailability and solubility of curcumin and achieve a sustained release, designing and developing appropriate drug carriers is critical. Here, porous vaterite CaCO_3_ particles are used as the carrier for controllable release of curcumin.

**Figure 15:**
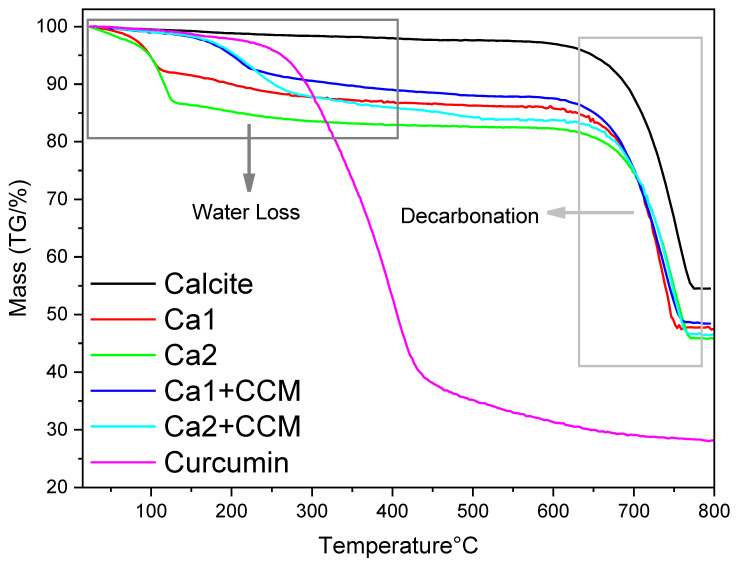
TGA profiles of calcite, curcumin (CCM), Ca 1, Ca 2,Ca 1 + CCM, Ca 2 + CCM particles.

The SEM images of curcumin-loaded particles (Figure S7) showed no visible curcumin precipitate on particle surface, indicating the successful encapsulation of curcumin within the vaterite particles. Furthermore, the TEM image of curcumin-loaded Ca 1 exhibited a denser structure than the blank Ca 1 particle (Figure S8), another indication of curcumin encapsulation. The thermogravimetric analysis (TGA) of the vaterite particles with loaded curcumin was recorded in the temperature range of 20 to 800 °C. As shown in Figure 15, all calcium carbonate particles exhibit similar behaviour within the 650–750 °C temperature range, which is the main mass loss attributing to the decomposition of CaCO_3_ and the removal of produced CO_2_. The TGA profile indicates that decarbonation begins after 600 °C. For pure calcite, there is a lower mass loss. The small mass loss from 25 to 600 °C is about 2% which could be the moisture absorbed in the powder. The endothermic decomposition of CO_2_ occurs between 620–780 °C, corresponding to the conversion of CaCO_3_ to CaO, resulting in a 40% mass loss (theoretical mass loss is around 44%). For Ca 1 and Ca 2, because Ca 1 has a small portion of calcite compared to Ca 2, the water content in Ca 1 is expected to be lower than in Ca 2. From the TGA profile, a mass loss of 8% is observed between 20–110 °C for Ca 1, 13% for Ca 2. A second mass loss of 5–10% is observed between 110–600 °C, corresponding to the elimination of water within the vaterite structure. For curcumin-loaded vaterite Ca 1 and Ca 2 particles, the mass loss in the temperature range of 20–110 °C looks lower, compared to blank Ca 1 and Ca 2 particles. This is likely due to the moisture content removed during the rotary evaporation process which was employed to prepare curcumin-loaded particles. However, a more significant mass loss is observed in the temperature range of 200–550 °C, which is attributed to the mass loss of curcumin during the heating procedure.

**Figure 16:**
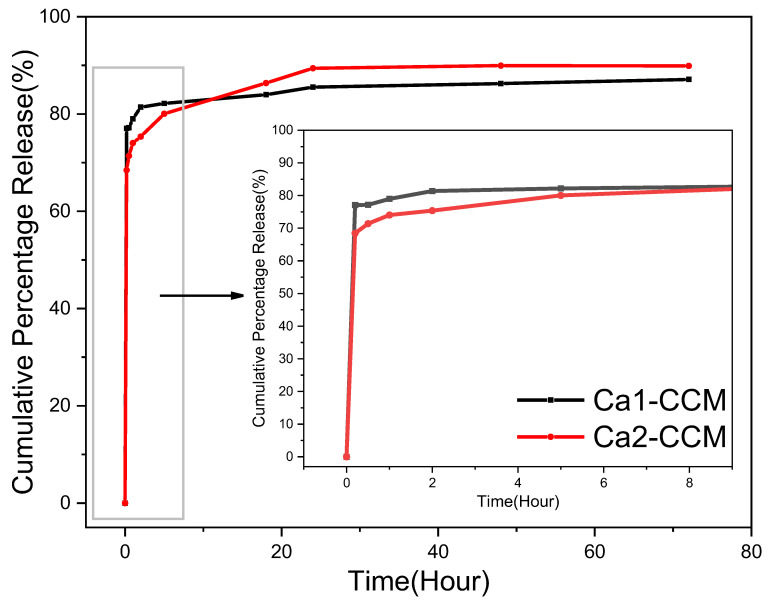
The release profiles of curcumin from curcumin-loaded particles Ca 1-CCM and Ca 2-CCM in a 1:1 *v*/*v* ethanol-phosphate buffer solution at room temperature.

The release study was carried out in the 1:1 PBS: ethanol solution to slow down and prevent the transformation of vaterite to calcite while ensuring the release of curcumin. Figure 16 illustrates the drug release profiles of curcumin-loaded Ca 1 and Ca 2. The release of curcumin was monitored by UV-vis spectroscopy and calculated based on the calibration curve of curcumin in 1:1 *v*/*v* PBS:ethanol solution (Figure S9). Both samples exhibit a sharp burst release within the first 10 min, followed by a steady and slow release. The high initial release can be attributed to the curcumin loaded on or close to the particle surface. Overall, vaterite Ca 2 achieves a 90% drug release, while Ca 1 reaches 87.11%. Ca 1, with larger particle size and small surface area, shows a higher initial drug release because more curcumin is deposited on the superficial surface of the structure. Ca 2 shows a lower initial burse release, and then slower and longer release of more curcumin. This results from the smaller particle size, higher surface area, and highly interconnected porosity. This demonstrates that it is possible to control and tune the drug release profile by carefully selecting conditions for the preparation of carrier CaCO_3_ particles.

## 4. Conclusions

A systematic investigation on the formation of porous vaterite CaCO_3_ particles under different conditions has been carried out. By altering preparation parameters such as salt precursor, salt concentration, salt ratio, reaction temperature, and the concentration of additive ethylene glycol, we have demonstrated how these parameters could influence the polymorph and morphology of the formed CaCO_3_ particles. Ethylene glycol could induce the supersaturation ratio and promote the formation of vaterite particles. The porosity of the CaCO_3_ particles could be adjusted by the reaction temperature, where higher temperatures facilitated the formation of larger primary nanocrystallites, leading to low specific surface area and low porosity of the secondary vaterite particles. The reaction conditions could be optimized to prepare vaterite nanoparticles (~200 nm) with a high porosity. The porous vaterite nanoparticles were further used as a drug carrier. The controlled release of a model drug curcumin was demonstrated with two types of vaterite particles, indicating how preparation conditions could be adjusted to prepare CaCO_3_ particles with different characteristics to suit different types of target applications.

## Figures and Tables

**Table 1:** Conditions used for the synthesis of CaCO_3_ particles.

Sample	Na_2_CO_3_		NaHCO_3_		CaCl_2_		Ca(NO_3_)_2_		Reaction Temp (°C)	Ethylene Glycol (*v*/*v*%)
	Conc. (M)	mL	Conc. (M)	mL	Conc. (M)	mL	Conc. (M)	mL		
S1	0.5	3			0.5	3			20	0
S2	0.5	3			0.5	3			20	15
S3	0.5	3			0.5	3			20	50
S4	0.5	3			0.5	3			20	85
S5	0.5	20			0.5	20			0	0
S6	0.5	20			0.5	20			20	85
S7	0.1	3			0.1	3			20	85
S8	0.1	3			0.1	3			60	85
S9	0.05	3			0.05	3			0	85
S10	0.05	3			0.05	3			20	85
S11	0.05	3			0.05	3			60	85
S12	0.05	3					0.05	3	20	85
S13			0.05	3			0.05	3	20	85
S14			0.05	3	0.05	3			60	85
S15			0.05	6	0.05	3			20	85
S16	0.5	6					0.5	3	20	85
S17	0.05	6			0.05	3			20	85
S18	0.1	6			0.1	3			60	85
S19			0.1	6	0.1	3			60	85
S20			0.1	6			0.1	3	60	85
Vaterite Ca 1	0.5	20					0.5	20	20	50
Vaterite Ca 2	0.025	5			0.025	5			20	85

**Table 2:** Specific surface area, pore volume, and pore diamemeter of vaterite particles prepared from 0.05 M solutions with 85 *v*/*v*% at 0, 20, and 60 °C.

Sample	Specific Surface Area (m^2^/g)	Pore Volume (cm^3^/g)	Pore Diameter (nm)
S9	10.99	0.026	4.04
S10	17.54	0.038	4.17
S11	11.50	0.047	6.66

**Table 3:** Specific surface area, pore volume, and pore diamemeter measured by N_2_ sorption at 77.3 K for samples Ca 1 and Ca 2.

Sample	Specific Surface Area (m^2^/g)	Pore Volume (cm^3^/g)	Pore Diameter (nm)
Ca 1	5.32	0.024	7.91
Ca 2	20.40	0.1420	11.93

## Data Availability

Dataset available upon request from the authors. The raw data supporting the conclusions of this article will be made available by the authors upon request.

## Supplementary Materials

The following supporting information can be downloaded at https://www.mdpi.com/article/10.3390/nano15161227/s1. Figure S1: FTIR spectra of calcium carbonate phase transformation at different reaction times. Figure S2: The SEM images of the porous structure of vaterite particles prepared from 0.05 M solutions with 85 *v*/*v*% at 20 °C and 60 °C. Figure S3: The Size distribution of vaterite particles obtained from the DLS analysis for the same samples in Figure 10. Figure S4: The FTIR of vaterite formed at a Ca^2+^:CO_3_^2−^ ratio of 1:1 for the same samples in Figure 10. Figure S5: FTIR spectra of CaCO_3_ particles formed at a Ca^2+^:CO_3_^2−^ ratio of 1:2 under the conditions used in Figure 11. Figure S6: FTIR and XRD results of sample Ca 2. Figure S7: SEM images of curcumin-loaded Ca and vaterite Ca 2 particles. Figure S8: TEM images of a curcumin-loaded and blank Ca 1 particle. Figure S9: Calibration curve of curcumin in 1:1 volume ratio of ethanol to PBS.
