# Supplementary material for: Systematic Study of Preparing Porous CaCO3 Vaterite Particles for Controlled Drug Release
Source: Nanomaterials (Basel). 2025 Aug 12;15(16):1227. doi: 10.3390/nano15161227 (PMC12388638; doi:10.3390/nano15161227)
Supplement: Supplementary file 1 [file nanomaterials-15-01227-s001.zip › nanomaterials-3770963-supplementary.pdf]

Systematic Study of Preparing Porous $CaCO_3$ Vaterite Particles for Controlled Drug Release

Nan Zhang, Binhang Zhao, Pan Yang, Haifei Zhang*

Department of Chemistry, University of Liverpool, Liverpool, L69 7ZD, UK

*Correspondence: zhanghf@liverpool.ac.uk.

Supporting Information

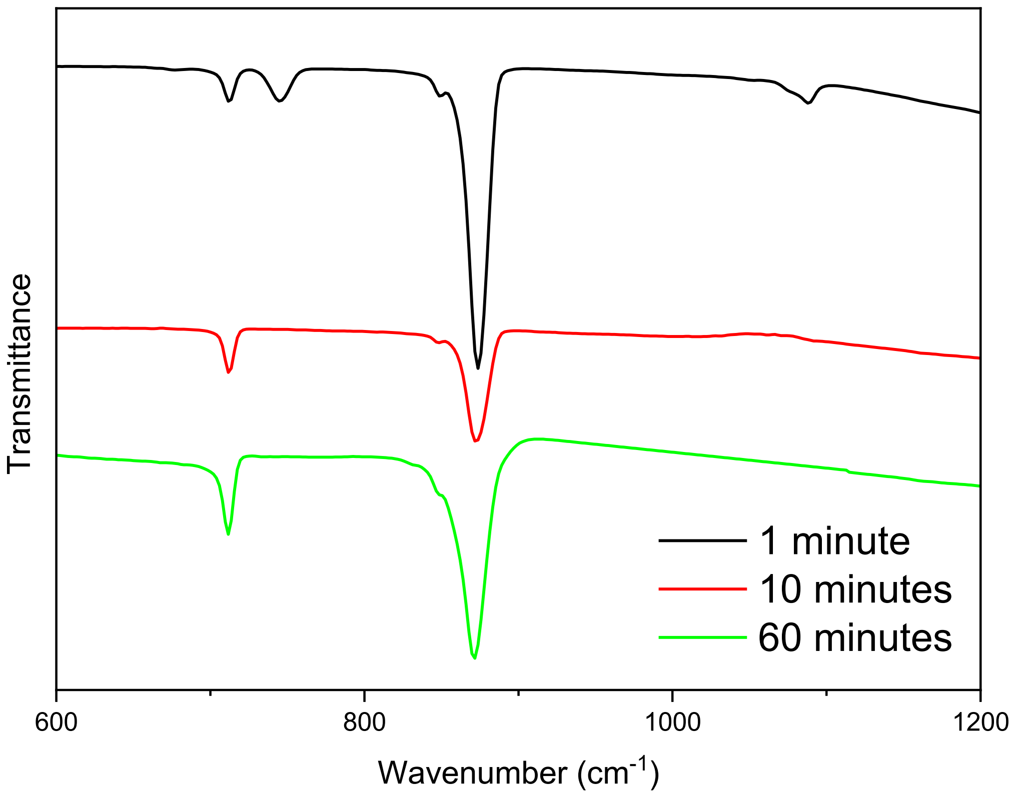

Fig. S1. FTIR spectra of calcium carbonate phase transformation, Na_2CO_3 solution (0.5 M, 20 ml) was rapid mixed with $CaCl_2$ solution (0.5 M, 20ml) in the ice bath. The sample was collected at (A) 1 mins (B) 10mins (C) 60mins.

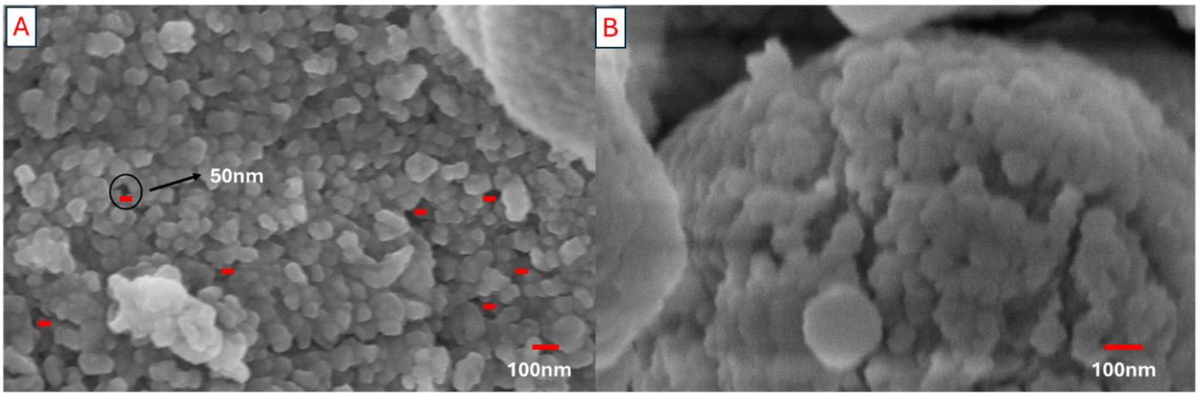

Fig. S2. The SEM images of the porous structure of vaterite particles prepared from 0.05 M solutions with 85 v/v% at (A) 20 ℃ and (B) 60 ℃.

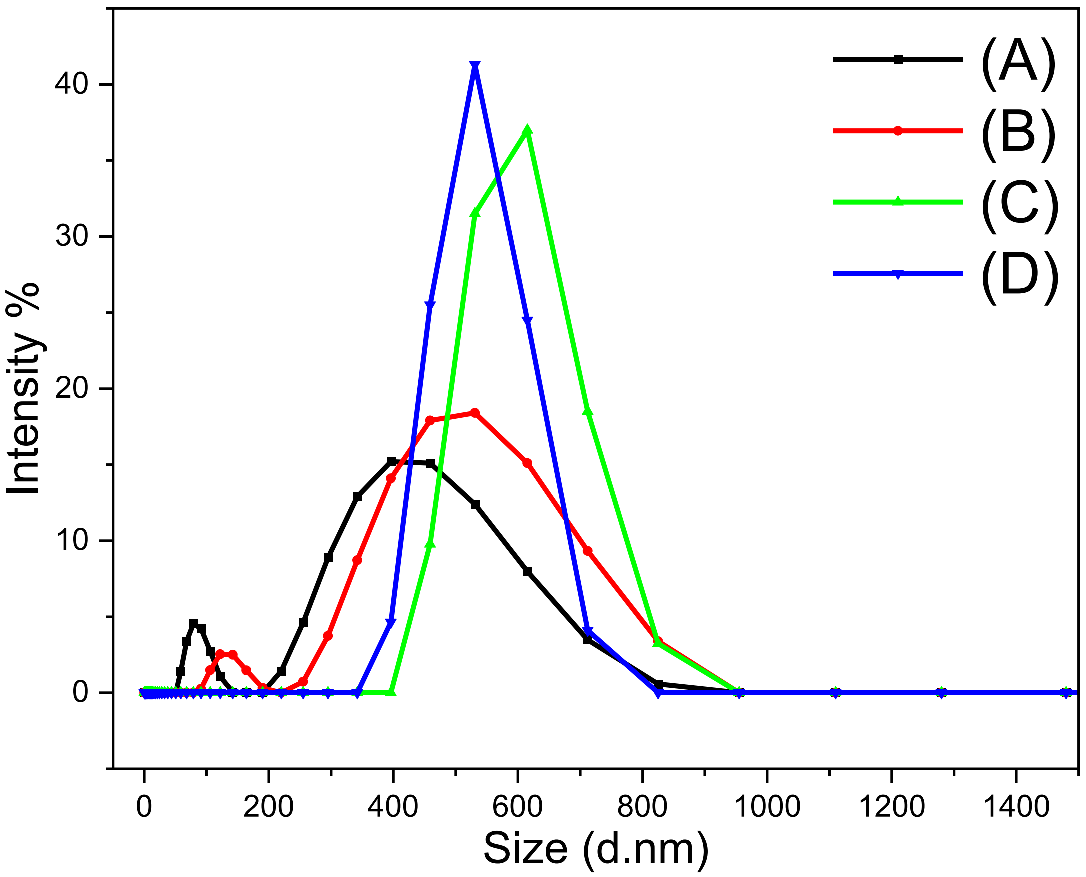

Fig. S3. The Size distribution of vaterite particles obtained from the DLS analysis that formed at a Ca^{2+}:CO_3^{2-} ratio of 1:1 in Fig. 10. (A) 0.05M of Na_2CO_3: $Ca(NO_3)_2$ at 20 °C, (B) 0.05M of $CaCl_2$:Na_2CO_3 at 20 °C, (C) 0.05M of $Ca(NO_3)_2$, $NaHCO_3$ at 20 °C, (D) 0.05M of $CaCl_2$, $NaHCO_3$ at 60°C.

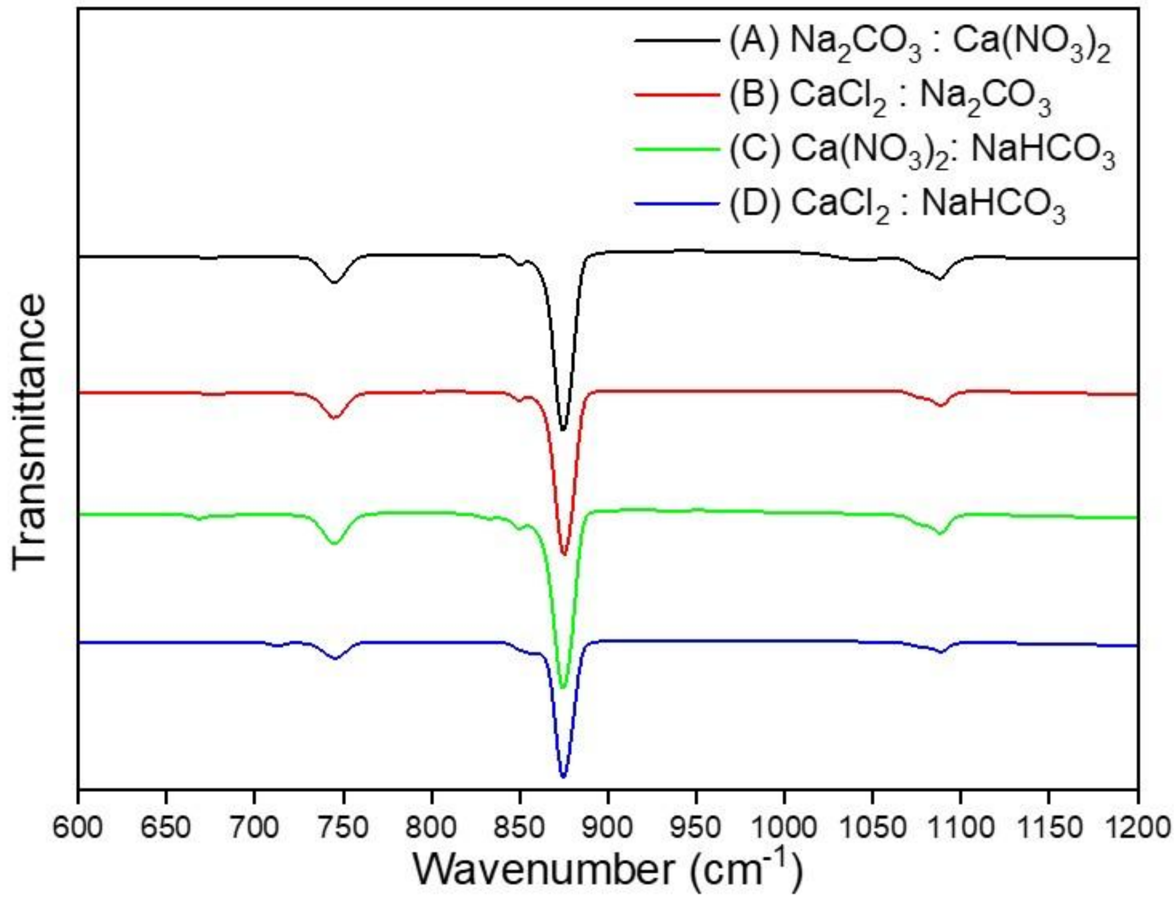

Fig. S4. The FTIR of vaterite formed at a Ca^{2+}:CO_3^{2-} ratio of 1:1 in Fig. 10. (A) 0.05M of Na_2CO_3: $Ca(NO_3)_2$ at 20 °C, (B) 0.05M of $CaCl_2$:Na_2CO_3 at 20 °C, (C) 0.05M of $Ca(NO_3)_2$, $NaHCO_3$ at 20 °C, (D) 0.05M of $CaCl_2$, $NaHCO_3$ at 60°C.

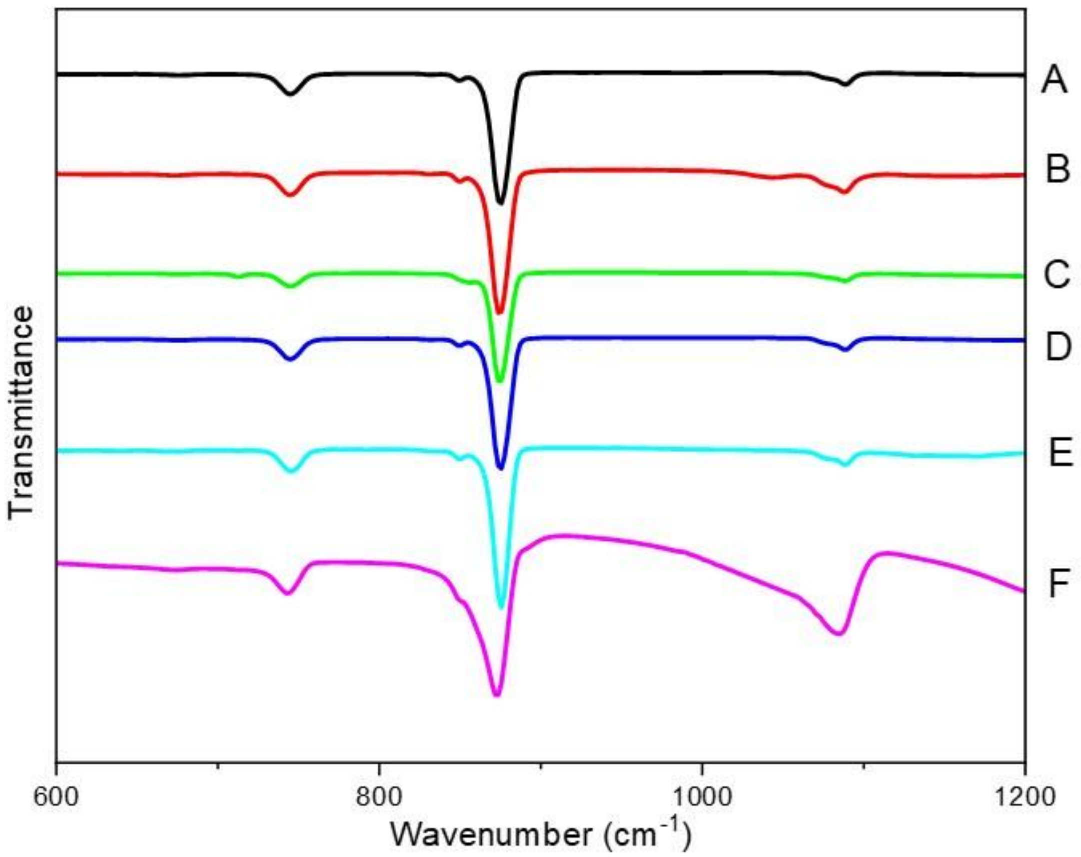

Fig. S5. FTIR spectra of $CaCO_3$ particles formed at a Ca^{2+}:CO_3^{2-} ratio of 1:2 under the conditions used in Fig. 11. (A) 0.05M of $CaCl_2$, $NaHCO_3$ at 20 °C. (B) 0.5M of Na_2CO_3, $Ca(NO_3)$2 at 20 °C, (C) 0.05M of $CaCl_2$, Na_2CO_3 at 20 °C, (D) 0.1M of $CaCl_2$, Na_2CO_3 at 60°C, (E) 0.1M of $CaCl_2$, $NaHCO_3$ at 60°C, (F) 0.1M of $Ca(NO_3)_2$, $NaHCO_3$ at 60°C.

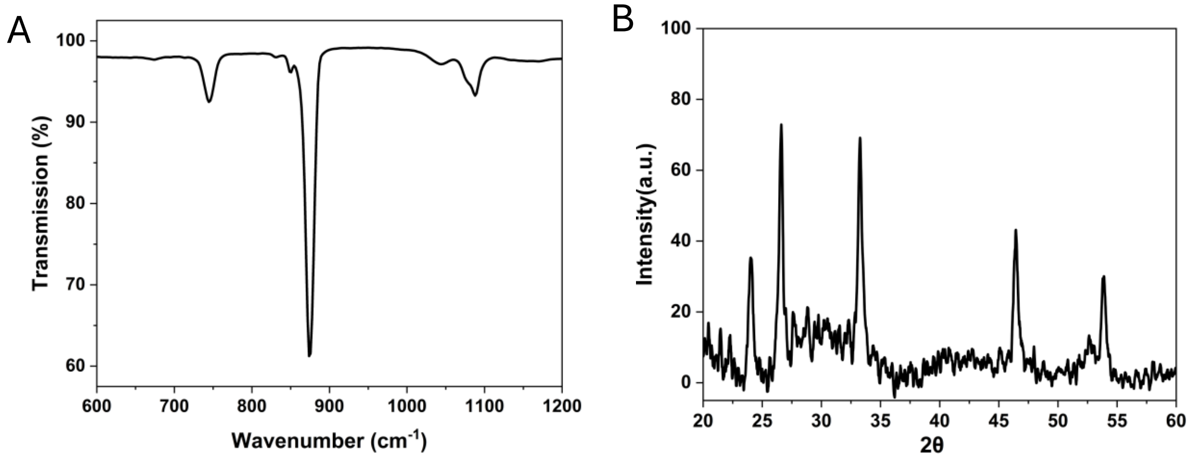

Fig. S6. FTIR spectrum (A) and PXRD diffractogram (B) of sample Ca 2.

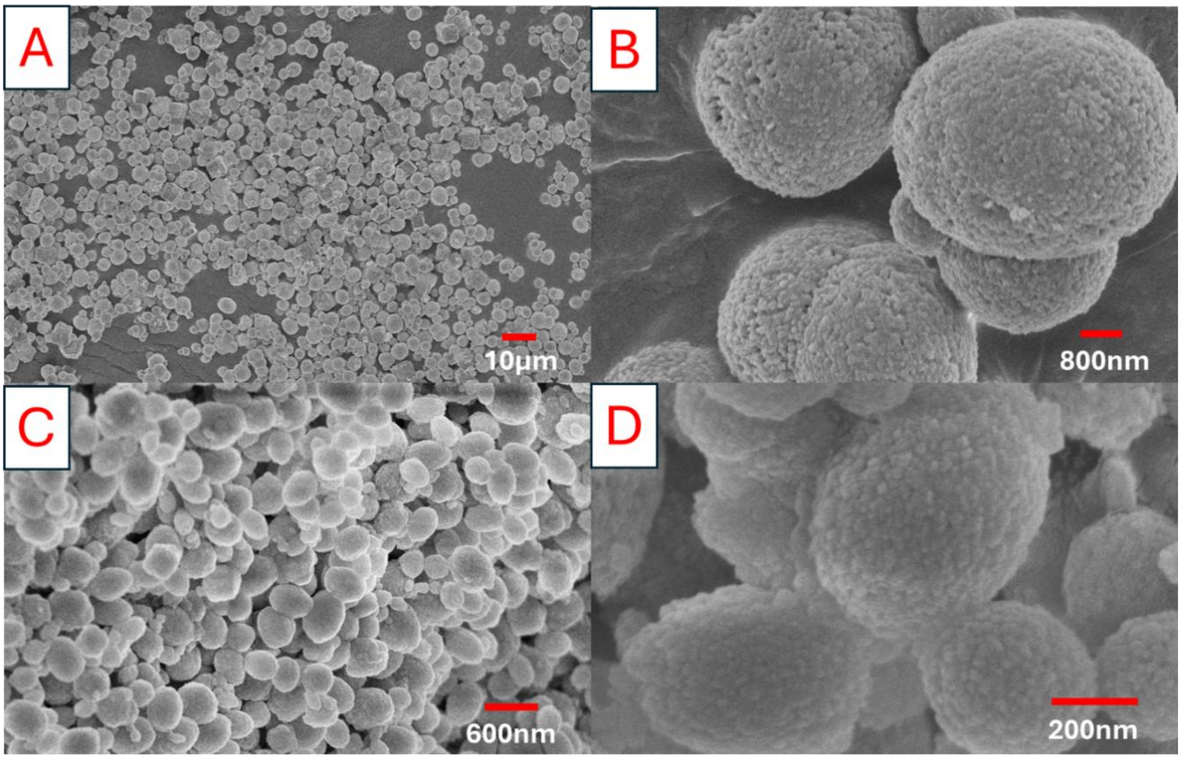

Fig. S7. SEM images of curcumin-loaded Ca 1 (A-B) and vaterite Ca 2(C-D) particles.

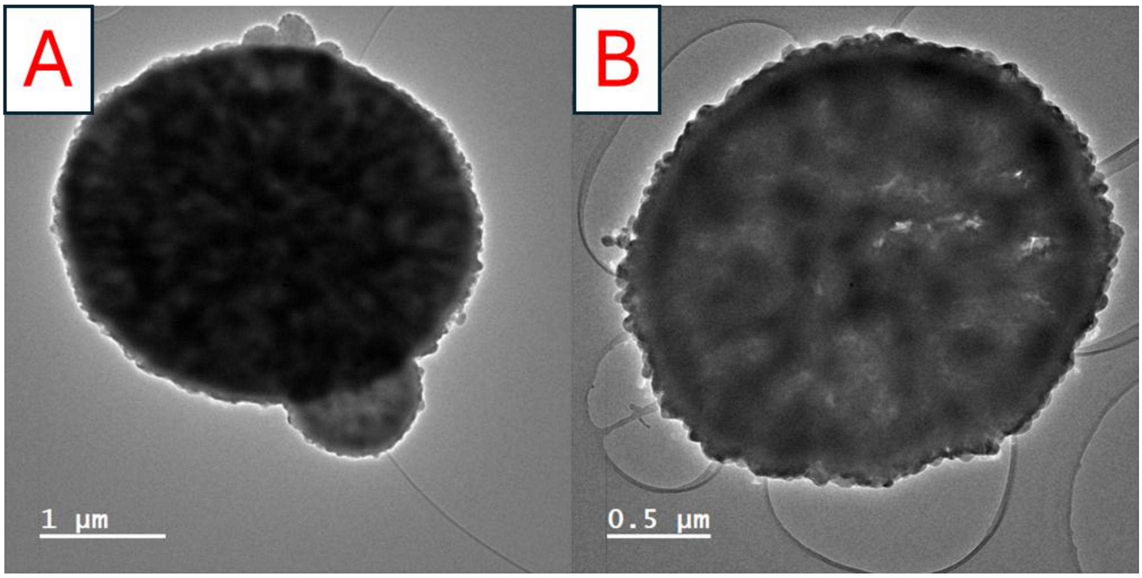

Fig. S8. (A) TEM image of a curcumin-loaded Ca 1 particle. (B)TEM image of the blank Ca 1 particle before loading with curcumin.

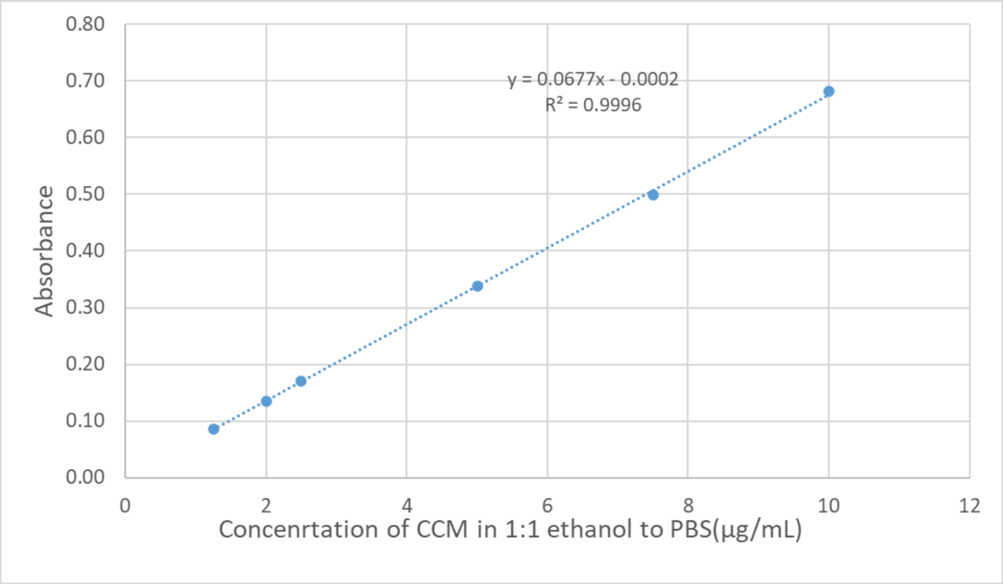

Fig. S9. Calibration curve of curcumin in 1:1 volume ratio of ethanol to PBS.
